# Supplementary material for: Dual Binding of an Antibody and a Small Molecule Increases the Stability of TERRA G-Quadruplex
Source: Angew Chem Int Ed Engl. 2014 Nov 24;54(3):910–3. doi: 10.1002/anie.201408113 (PMC4506565; doi:10.1002/anie.201408113)
Supplement: Supplementary file 1 [file anie0054-0910-sd1.pdf]

Angewandte Chemie
Eine Zeitschrift der Gesellschaft Deutscher Chemiker

Supporting Information
© Wiley-VCH 2014
69451 Weinheim, Germany

Dual Binding of an Antibody and a Small Molecule Increases the Stability of TERRA G-Quadruplex**

Philip M. Yangyuoru, Marco Di Antonio, Chiran Ghimire, Giulia Biffi, Shankar Balasubramanian, and Hanbin Mao**

anie_201408113_sm_miscellaneous_information.pdf

Supporting Information

Materials and Methods

Materials

Small molecule ligand carboxypyridostatin (cPDS) was synthesized as previously described.[1] The antibody (BG4) was prepared as previously described.[2] Aqueous stock solution (10 mM) of cPDS was stored at -80 °C, 1 mM working aliquots were stored at -20 °C, and BG4 was stored at 4 °C. DNA oligonucleotides were purchased either from Integrated DNA Technologies (IDT) (http://www.idtdna.com) or Gene Link (http://www.genelink.com) and further purified by denaturing polyacrylamide gel and stored at -20 °C. Cloned plasmid DNA template for *in vitro* RNA transcription was sequenced at the University of Maine DNA sequencing facility. TERRA synthetic oligonucleotide for ELISA binding assay was purchased from Sigma Aldrich.

TERRA Construct

The DNA-RNA hybrid construct was prepared by inserting the encoding TERRA quadruplex-forming sequence (5'- TTAGGG TTAGGG TTAGGG TTAGGG TTA -3') and cloning into a pBR322 plasmid (NEB, Ipswich, MA) between EcoRI and HindIII sites. The template for *in vitro* RNA synthesis (MEGAscript, Invitrogen) was generated from PCR amplification of the cloned plasmid (between the 3836th and 1473rd nucleotides) using primers containing the T7 promoter. Handle A (527 bp) was obtained from PCR amplification of the unmodified pBR322 plasmid (between the 3836th and 1st nucleotides) with an EcoRI digestion site on the forward primer, followed by EcoRI (NEB, Ipswich, MA) digestion and end-labeling

with DNA Polymerase I, Large Fragment (Klenow, NEB) in the presence of digoxigenin (Dig)-11-dUTP (Roche, Indianapolis, IN). Handle B (1473 bp) was prepared from PCR amplification of the unmodified pBR322 plasmid (between the 31st and 1473th nucleotides) with a 5'-biotin labeled reverse primer. The control construct was prepared similarly by replacing the TERRA sequence with the wild type sequence between the EcoRI and HindIII sites of pBR322.

The DNA-RNA hybrid constructs were prepared by annealing the RNA prepared by using MEGAscript® T7 high yield transcription kit (Ambion) and the two DNA handles via heating at 95 °C for 5 min and then gradually cooling down to room temperature for 4 h.

Single-Molecule Experiments

Laser tweezers experiments were performed by attaching the DNA-RNA hybrid constructs (~0.25 ng/µL) to the anti-Dig antibody coated polystyrene beads (diameter 2.10 µm, Spherotech, Lake Forest, IL) via the Dig/anti-Dig linkage for 30 min at room temperature. These beads and the streptavidin coated beads (1.87 µm, Spherotech, Lake Forest, IL) were injected into a microfluidic chamber and dispersed into a 1 mL 10 mM Tris buffer with 100 mM KCl, 1 mM EDTA and 1.5 µL RNase inhibitor (RNAsecure, Invitrogen) at pH 7.4. The buffer contains 5 µM cPDS and/or 50 nM BG4. The two different beads were separately trapped by two laser foci in a house-made laser tweezers instrument.[3] The DNA-RNA construct was tethered between the two trapped beads by moving the trapped beads together. Each tethered construct was repeatedly stretched and relaxed by force-ramping between 0 and 60 pN at a loading rate of 5.5 pN/s to record the force-extension (*F-X*) curves at 1000 Hz using Labview® program. Histograms of ΔL and rupture force were prepared by measuring 470 total molecules with and without ligands. Variation between individual molecules is not significant.

Single molecules were confirmed by a 65 pN plateau in rupture force observed for each *F-X* curve which indicates the denaturation of single dsDNA molecules under tension,[4] or single breakage of the tethered molecule to 0 pN. Accurate distance measurement of the instrument was confirmed by determining the contour length per DNA nucleotide. First, *F-X* curves of a DNA construct that contains a DNA hairpin, 5'-GC$(T)_{10}$GC<u>TTTT</u>GC$(A)_{10}$GC-3', with a tetrathymine loop (underscored), sandwiched between two dsDNA handles as described in literature,[5] were obtained. These *F-X* curves were then fitted into a sequential model[5] that uses two Worm Like Chain (WLC) functions[4] to represent the ssDNA hairpin and dsDNA handles. The WLC model is expressed as $\frac{x}{L}=1-\frac{1}{2}(\frac{k_BT}{FP})^{\frac{1}{2}}+\frac{F}{S}$, in which x is the end-to-end distance at a particular force (F), L is the contour length, k_B is the Boltzmann constant, T is absolute temperature, P is the persistence length, and S is the stretch modulus. A value of 0.44 ± 0.02 nm per DNA nucleotide was obtained, which is identical to that measured by Block and coworkers[6] and within the same range as determined by others.[7-10] For the TERRA GQ construct, the values of P (10.6 ± 1.8 nm) and S (191 ± 61 pN) for the hybrid DNA/RNA handles were obtained from sequential fitting of the *F-X* curves.[5] These values are consistent with those reported in literature.[11]

Population Deconvolution with Nanometer Resolution (PoDNano)

This data analysis method is used to identify the predominant ΔL populations in a ΔL histogram.[12] First, the change-in-extension (Δx) for each *F-X* curve due to the unfolding of a secondary structure, sandwiched between two nucleic acid handles, was obtained at a particular force F from each set of unfolding and refolding traces. Average standard error (σ) values from the regions immediately after and before the rupture transitions were obtained for each ΔL.

Using this value as the width of a Gaussian kernel, a kernel density distribution was constructed for the ΔL of all unfolding transitions. The most probable populations in the constructed ΔL histogram were identified by performing 3,000 random resampling bootstrapping analyses. From each resampling, two highest peaks were identified by the Igor® program (WaveMatrics, Portland, OR). These selected peaks were used to construct the PoDNano histograms.

Force-Jump Experiments

The detailed force-jump procedures have been described elsewhere.[13] Briefly, the TERRA construct tethered between the two beads as stated above was stretched until the structure was unfolded, then relaxed to 0 pN in 10 ms, followed by incubation for varied time durations (0-180 s) to monitor the refolding of the structure. Rupture events in the subsequent unfolding curves that start at 10 pN by another force jump indicate the refolding of secondary structures during the incubation.

The TERRA GQ formation probability with or without ligand for each incubation time was calculated as the ratio of the number of unfolding events observed in subsequent curves to the total subsequent pulling events (folded + unfolded).

Change in Free Energy of Unfolding (ΔG_{unfold})

Change in free energy for the mechanical unfolding of the TERRA structures ($\Delta G_{\mathrm{unfold}}$) were calculated by the Jarzynski equation for non-equilibrium systems,[14-15] $\Delta G_{\mathrm{unfold}} = -k_B T \ln \sum_{i=1}^{N} \frac{1}{N} \exp\left(-\frac{W_i}{k_B T}\right)$, where N is the number of observations and W is the non-equilibrium work done to unfold the structure(s), which is equivalent to the hysteresis area between unfolding and refolding F-X curves.

Fitting Equations for Ligand Binding Assays

To determine the folding rate constant, k_{fold}, from the kinetic measurements in the force-jump experiments with or without a ligand, the experimental data of the probability of unfolded and folded species were fitted into the two-state kinetic model shown below,[16]

$$\text{Unfolded (U)} \underset{k_{\text{unfold}}}{\overset{k_{\text{fold}}}{\rightleftharpoons}} \text{Folded (F)}$$

$$P_{U,t} = \frac{1}{k_1 + k_2}\left[k_2 + k_1 e^{-(k_1+k_2)t}\right] \ldots\ldots\ldots\ldots\ldots\ldots (1)$$

$$P_{F,t} = \frac{k_1}{k_1 + k_2}\left[1 - e^{-(k_1+k_2)t}\right] \ldots\ldots\ldots\ldots\ldots\ldots\ldots.. (2)$$

Here, P represents the folding probability, t is the incubation time, and k_{fold} and k_{unfold} are folding and unfolding rate constants, respectively. These equations were used to fit the refolding probability of GQ at different incubation times.

FRET-Melting Assays

100 μM stock solution of oligonucleotide was prepared in molecular biology grade DNase-free water. Further dilutions were carried out in 60 mM potassium cacodylate buffer, pH 7.4. FRET experiments were carried out with 200 nM oligonucleotide concentration. The labeled DNA oligonucleotide was supplied by IBA® GmbH (TERRA (5-FAM-GGG UUA GGG UUA GGG UUA GGG-TAMRA-3). The donor fluorophore was 6-carboxyfluorescein (FAM) and the acceptor fluorophore was 6-carboxytetramethylrhodamine (TAMRA). The dual-labeled

oligonucleotide was annealed at a concentration of 400 nM by heating at 94 ºC for 10 min followed by slow cooling to room temperature at a controlled rate of 0.1 ºC/min. 96-well plates were prepared by addition of 50 μL (400 nM) of the annealed DNA solution to each well, followed by 50 μL solution of cPDS (1 μM), BG4 (400 nM), or both at the same concentrations (1 μM cPDS and 400 nM BG4). Measurements were made in triplicate with an excitation wavelength of 483 nm and a detection wavelength of 533 nm.

Supporting Figures

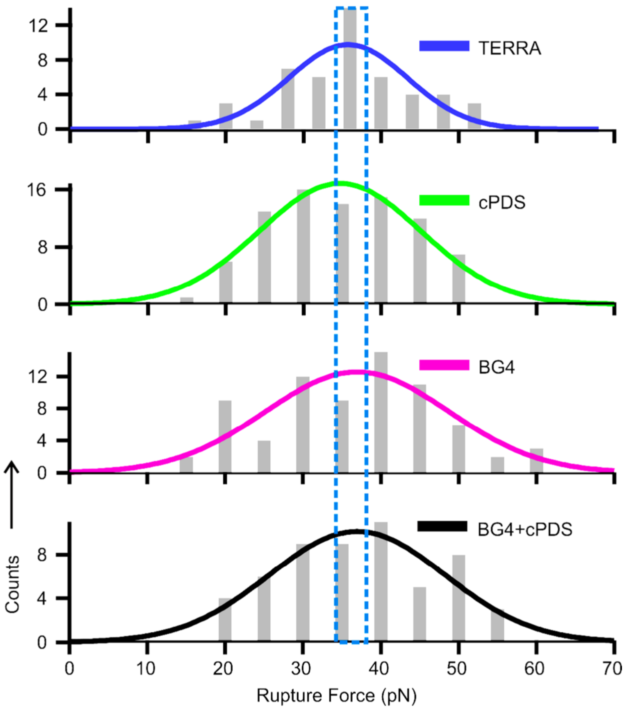

Figure S1: *Rupture force histograms of partially folded species (GT) obtained after 45-180 s incubation.*

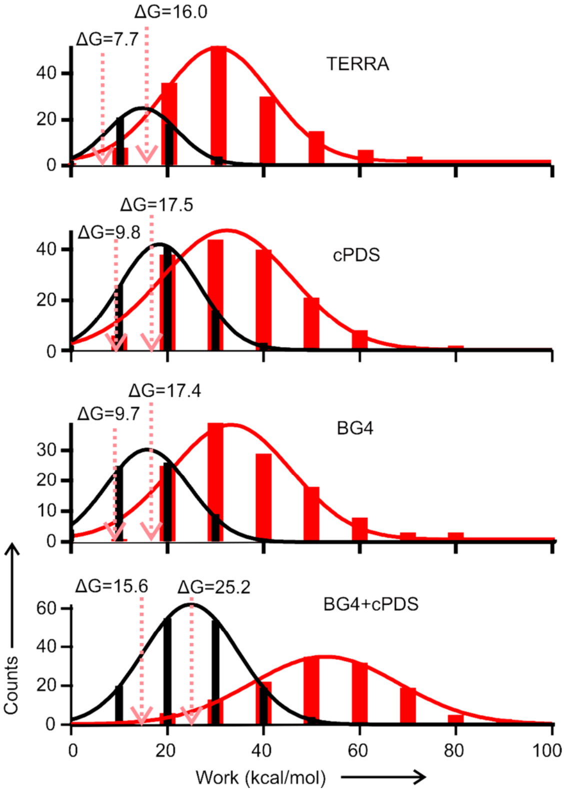

Figure S2: *Work histograms and* ΔG_{unfold} *calculation based on the small and big rupture force populations for TERRA G-quadruplex without ligand or in the presence of 5 µM cPDS, 50 nM BG4, or 5 µM cPDS + 50 nM BG4.*

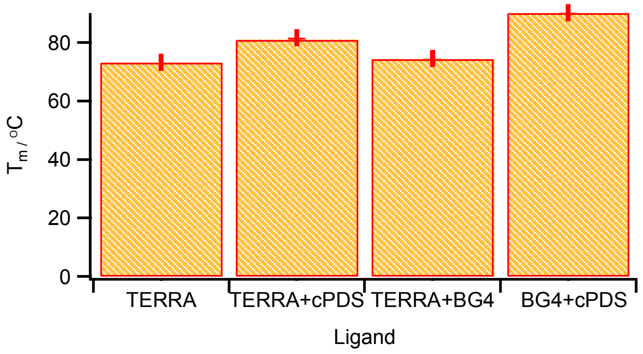

Figure S3: *FRET-melting assays. Bar chart diagram of FRET melting experiments (T_m) of a dually labeled TERRA G4 (200 nM) without ligand or in the presence of 500 nM cPDS, 200 nM BG4, or 500 nM cPDS + 200 nM BG4.*

Table S1: Change in the free energy of unfolding (ΔG_{unfold}) for the TERRA GQ bound with different ligands. The $\Delta\Delta G_{unfold}$ was calculated by the difference in the ΔG_{unfold} between ligand-bound and free TERRA G-quadruplexes for either the small or big rupture force population.

Ligand	TERRA		cPDS		BG4		cPDS+BG4	
Rupture force	Small (23 pN)	Big (36 pN)	Small (25 pN)	Big (40 pN)	Small (24 pN)	Big (40 pN)	Small (30 pN)	Big (50 pN)
% Formed	24	76	36	64	34	66	52	48
ΔG_{unfold} kcal/mol	8±2	16±3	10±2	17±1	10±4	17±2	15±1	25±3
$\Delta\Delta G_{unfold}$ kcal/mol			2.0±0.1	1.4±0.3	2.0±0.1	1.3±0.4	7.0±0.2	9.1±0.3

References

[1] M. Di Antonio, G. Biffi, A. Mariani, E. A. Raiber, R. Rodriguez, S. Balasubramanian, *Angew Chem Int Edit*. **2012**, *51*, 11073-11078.

[2] G. Biffi, D. Tannahill, J. McCafferty, S. Balasubramanian, *Nat. Chem.* **2013**, *5*, 182-186.

[3] H. Mao, P. Luchette, *Sensors and Actuators B*. **2008**, *129*, 764-771.

[4] S. B. Smith, Y. J. Cui, C. Bustamante, *Science*. **1996**, *271*, 795-799.

[5] W. J. Greenleaf, K. L. Frieda, D. A. Foster, M. T. Woodside, S. M. Block, *Science*. **2008**, *319*, 630-633.

[6] M. T. Woodside, W. M. Behnke-Parks, K. Larizadeh, K. Travers, D. Herschlag, S. M. Block, *Proc. Natl. Acad. Sci. U. S. A.* **2006**, *103*, 6190-6195.

[7] J. B. Mills, E. Vacano, P. J. Hagerman, *J. Mol. Biol.* **1999**, *285*, 245-257.

[8] B. Tinland, A. Pluen, J. Sturm, G. Weill, *Macromolecules*. **1997**, *30*, 5763-5765.

[9] M. T. J. Record, C. F. Anderson, T. M. Lohman, *Quart. Rev. Biophys.* **1978**, *11*, 103-178.

[10] T. A. Laurence, X. Kong, M. Jager, S. Weiss, *Proc. Nat. Acad. Sci. USA*. **2005**, *102*, 17348-17353.

[11] B. Onoa, S. Dumont, J. Liphardt, S. B. Smith, I. Tinoco, Jr., C. Bustamante, *Science*. **2003**, *299*, 1892-1895.

[12] Z. Yu, H. Mao, *Chemical Record*. **2013**, *13*, 102-116.

[13] D. Koirala, S. Dhakal, B. Ashbridge, Y. Sannohe, R. Rodriguez, H. Sugiyama, S. Balasubramanian, H. Mao, *Nat. Chem.* **2011**, *3*, 782-787.

[14] J. Liphardt, S. Dumont, S. B. Smith, I. Tinoco, Jr., C. Bustamante, *Science*. **2002**, *296*, 1832-1835.

[15] C. Jarzynski, *Phys. Rev. Lett.* **1997**, *78*, 2690 - 2693.

[16] D. Koirala, C. Ghimire, C. Bohrer, Y. Sannohe, H. Sugiyama, H. Mao, *J. Am. Chem. Soc.* **2013**, *135*, 2235-2241.
